# 3D printable and myoelectrically sensitive hydrogel for smart prosthetic hand control

**DOI:** 10.1038/s41378-024-00825-y

**Published:** 2025-01-21

**Authors:** Jinxin Lai, Longya Xiao, Beichen Zhu, Longhan Xie, Hongjie Jiang

**Affiliations:** Shien-Ming Wu School of Intelligent Engineering, South China University of Technology, Guangzhou, 511442 P. R. China

**Keywords:** Physical sciences, Chemistry

## Abstract

Surface electromyogram (sEMG) serves as a means to discern human movement intentions, achieved by applying epidermal electrodes to specific body regions. However, it is difficult to obtain high-fidelity sEMG recordings in areas with intricate curved surfaces, such as the body, because regular sEMG electrodes have stiff structures. In this study, we developed myoelectrically sensitive hydrogels via 3D printing and integrated them into a stretchable, flexible, and high-density sEMG electrodes array. This electrode array offered a series of excellent human-machine interface (HMI) features, including conformal adherence to the skin, high electron-to-ion conductivity (and thus lower contact impedance), and sustained stability over extended periods. These attributes render our electrodes more conducive than commercial electrodes for long-term wearing and high-fidelity sEMG recording at complicated skin interfaces. Systematic in vivo studies were used to investigate its efficacy to control a prosthetic hand by decoding sEMG signals from the human hand via a multiple-channel readout circuit and a sophisticated artificial intelligence algorithm. Our findings demonstrate that the 3D printed gel myoelectric sensing system enables real-time and highly precise control of a prosthetic hand.

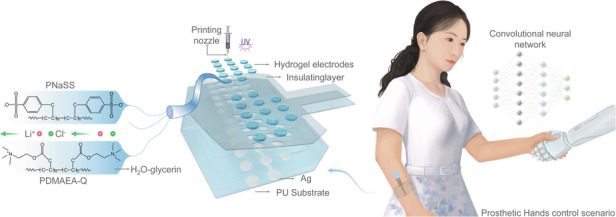

## Introduction

Numerous soft electronic devices have been developed in recent years^1–5^, which offer a range of capabilities including biosignal detection^4,6^, health monitoring^7,8^, nerve stimulation^1,9–11^, brain–computer interfaces^12^, neuromuscular junctions^13^ and cyborg tissues^14–16^. So, soft and stretchable bioelectronics that are in close contact with biological tissue interfaces have attracted increasing attention in the scientific community^17–19^. Bioelectronics has the capability to capture both electrophysiological and physical signals, including vital signs like heart rates, body motions, and tissue deformations. These signals can be utilized for diagnosing and managing a wide array of medical conditions. For example, a neural microelectrode array (MEA) can record electroencephalogram (EEG), electromyography (EMG), or electrocardiogram (ECG) signals of live tissues, which can be used to diagnose and treat various nervous system diseases, such as epilepsy or Parkinson’s disease^20–24^. Studies have demonstrated that electrophysiological signals like EMG or ECG can be utilized to identify human movement intentions, acquired by positioning epidermal electrodes to targeted areas of the body^25,26^. These signals are ahead of the limb movement in the time. Among them, EMGs are more suitable for usage in motion recognition because they are easier to collect and process^27,28^. However, the diverse nature of muscles, signal amplitudes ranging from microvolts to millivolts, and varying detection sites pose challenges. Commercial electrodes with high modulus and low extensibility struggle to establish conformal contact with the skin, leading to a significant deterioration in stimulation and recording performance, thereby posing a notable challenge to sEMG signal acquisition^29,30^.

In recent years, several efforts have been invested in optimizing HMI to boost the stimulation or recording properties of epidermal electrodes^31,32^. Plenty of on-skin electronics with stretchable conductive materials^33–35^ and flexible substrates^36,37^ have been developed. However, these flexible patches still present higher modulus than skin and have low adhesion, which obstructs the maintenance of seamless and stable contact between the device and the skin. Lately, hydrogels have garnered significant attention as promising interface materials for flexible electronics, owing to their advantageous characteristics such as remarkable stretchability, adhesion, low modulus, and tissue-like properties^38,39^. In contrast to conventional rigid electronics that are physically and mechanically dissimilar to biological tissues, conductive hydrogel electronics offer both favorable electrical properties and ideal interfaces with tissues, thus mitigating issues like immunological responses or electrochemical instability resulting from significant mechanical disparities^40–42^. When combined with self-adhesive conductive hydrogel interface layers, on-skin devices can establish robust and conformal connections with the skin, enhancing charge injection capacity and sensing efficiency^43^. Although several responsive hydrogels have been proposed to address the challenge of achieving close contact between bioelectronics and biological tissue interfaces^44,45^, these approaches still exhibit certain limitations, including inadequate original adhesion, lengthy preparation time, lack of customizability, slow responsiveness, and limited adjustment range. Overall, the development of on-skin devices that enable efficient regulation of mechanical properties, electrical stimulation, and acquisitions of long-lasting and high-fidelity electromyography signals remains an ongoing pursuit.

**Fig. 1:**
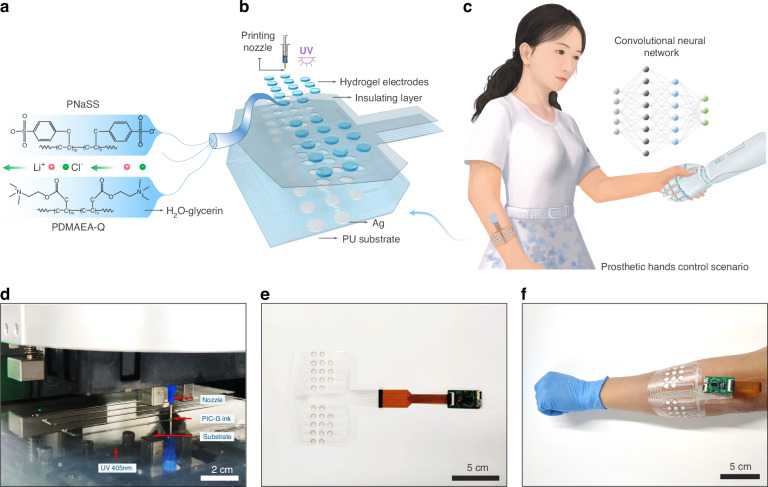
3D printing PIC-G hydrogels with tunable myoelectric sensing and mechanical properties for controlling prosthetic hand. **a** Schematic illustration of PIC-G gel structure. **b** The layer-by-layer construction of sEMG electrodes array. **c** Conceptual demonstration of the sEMG patch for the smart control of prosthetic hand. **d** Photograph showing the process of 3D printing PIC-G hydrogels. **e** Photograph showing the integrated sEMG system and **f** its practical application of wearing on the hand for recognition of hand gestures

To address these limitations, we propose a 3D printed polyion complex glycerol (PIC-G) hydrogel-dependent high-density sEMG electrode array, which is stretchable and robust enough to be worn on complicated skin interfaces (Fig. 1). Relying on the ionic bonding of PIC-G gels, a prepared hydrogel ink can be converted into a programmable 3D gel by chemically or physically gelating it under a UV light. This process allows for the creation of a semi-interpenetrating polymer network (semi-IPN) hydrogel, comprising a first dense network (poly (sodium p-styrenesulfonate) PNaSS) interpenetrated by a secondary loose network (poly (dimethylaminoethylacrylate quaternized ammonium) PDMAEA-Q). Moreover, the electrical and mechanical of the resultant hydrogels can be tuned concurrently through ink formulations or cross-linking conditions, for instance, obtaining a contact impedance of 10 kΩ with a Young’s modulus of 0.04 MPa (much lower than that of human skin, which ranges from 0.5 to 1.95 MPa). Owing to their compactness and lightweight, sEMG electrodes based on those hydrogel, incorporated with wireless readout capabilities, can be applied to target muscles to significantly improve the detection accuracy when recognizing hand gestures, which typically require a large number of conventional wired electrodes. To demonstrate, the patch was first attached to a group of specific hand muscles to obtain their sEMG signals during the flexion of each digit. Following analysis by a well-trained deep learning algorithm, the sEMG data were processed as inputs for the real-time classification of each digit’s movement and, ultimately, for the control of a prosthetic hand. Collective results illustrate how printed electronics integrated with soft materials can advance human performance and healthcare as a result of the proposed materials optimization, device integration, and sEMG-based HMIs.

## Results and discussion

### Design and implement of PIC-G ink

Utilizing 3D printable ink for device fabrication effectively mitigates the drawbacks of conventional gelation techniques. Traditional methods often demand substantial quantities of liquid precursors for in situ solidification, leading to significant waste of both resources and energy, while also resulting in inconsistent gelation and inadequate control over material characteristics. In addition, these traditional techniques struggle with the precise patterning of intricate microstructures due to their dependence on molds with grooves. In contrast, 3D printing facilitates enhanced geometric precision, swift customization, and the capability to construct complex structures, thereby improving the functionality of soft electronics through thoughtful structural design. The PIC-G ink, which exhibits excellent rheological properties, is synthesized through the stepwise polymerization of cationic and anionic monomers (see “Experimental section” for a detailed process of preparing PIC-G inks). Under shear forces, they behave as low-viscosity fluids, which facilitates extrusion printing^46^. However, once the external force is removed, the hydrogels quickly revert to a state of high viscoelasticity, ensuring the stability and accuracy of the printed structure’s shape. This characteristic is particularly advantageous for 3D printing complex and delicate structures. In addition, the material itself possesses excellent biocompatibility and tunability, allowing PIC hydrogels to meet diverse application requirements in terms of mechanical strength, flexibility, and elasticity, especially in complex design scenarios encountered in 3D printing.

As shown in Fig. 1a, the PIC hydrogel is composed of a network formed by the interaction of polyanion (PNaSS) and polycation (PDMAEA-Q), cross-linked under UV irradiation with a photoinitiator (Lap). To regulate ink viscosity and printability, an aqueous glycerin solution is incorporated into the ink formulation. In addition, incorporating glycerol into the ink formulation effectively enhances the mechanical properties and water retention of the gel. The addition of lithium chloride improves the electrical performance of the gel, making it a well-rounded choice for 3D printing applications. As a bifunctional cross-linking agent, MBAA reacts with anionic monomers, forming a three-dimensional cross-linking network to enhance the mechanical strength and stability of hydrogels. Therefore, optimal concentrations of photoinitiator, cross-linker, and UV irradiation time are crucial for ensuring an efficient and homogeneous cross-linking reaction, ensuring uniform ink distribution and consistent electrical properties across the gel structure. To validate the uniformity of the PIC-G ink, trips of varying lengths were 3D printed using the ink, and their resistances were measured. The findings revealed a highly linear and proportional relationship between the lengths of hydrogel strips and their electrical resistances. For instance, when the lengths of the strips ranged from 1 to 8 cm, the resistances of the hydrogel strips exhibited variation from 38.8 to 318.4 kΩ (Fig. S1).

The hydrogel-based sEMG electrodes patch (Fig. 1b) consists of four main parts, featuring a layer-by-layer lamination technique. From bottom to top, these layers are as follows: (1) a thin elastomeric polyurethane (PU) film that serves as the substrate for the sEMG electrodes, (2) a layer of screen printed silver (Ag) conductive electrodes that serves as the signal communication, (3) a group of PIC-G hydrogels (0.6 cm diameter) 3D printed on top of Ag electrodes that combine to form sEMG electrodes, and (4) a double adhesive PU layer that encapsulates the device and interfaces with the skin. Incorporating a wireless readout circuit, the device can be positioned comfortably on curved, soft skin, facilitating the transmission of sEMG information from body to the computer or machine in a more natural and undisturbed manner. A prime location for such placement is the human forearm, where the system can capture and interpret sEMG signals generated during a handshake gesture, using them as feedback to manipulate a robotic arm for a self-handshake (Fig. 1c). Notably, leveraging existing printing technologies streamlines the overall electrode manufacturing process, making it both straightforward and scalable. For instance, PIC-G gels and Ag electrodes can be rapidly patterned in sequence on a flexible substrate using a commercial inkjet printer (Fig. 1d), leaving an electrical interface for connecting to a multi-channel readout via a FPC (flexible printed circuit) connector (Fig. 1e). Consequently, this integrated system, coupled with a miniaturized external circuit, affords a flexible, stretchable, and wearable design (Fig. 1f).

### Rheological and mechanical properties of PIC-G gel

**Fig. 2:**
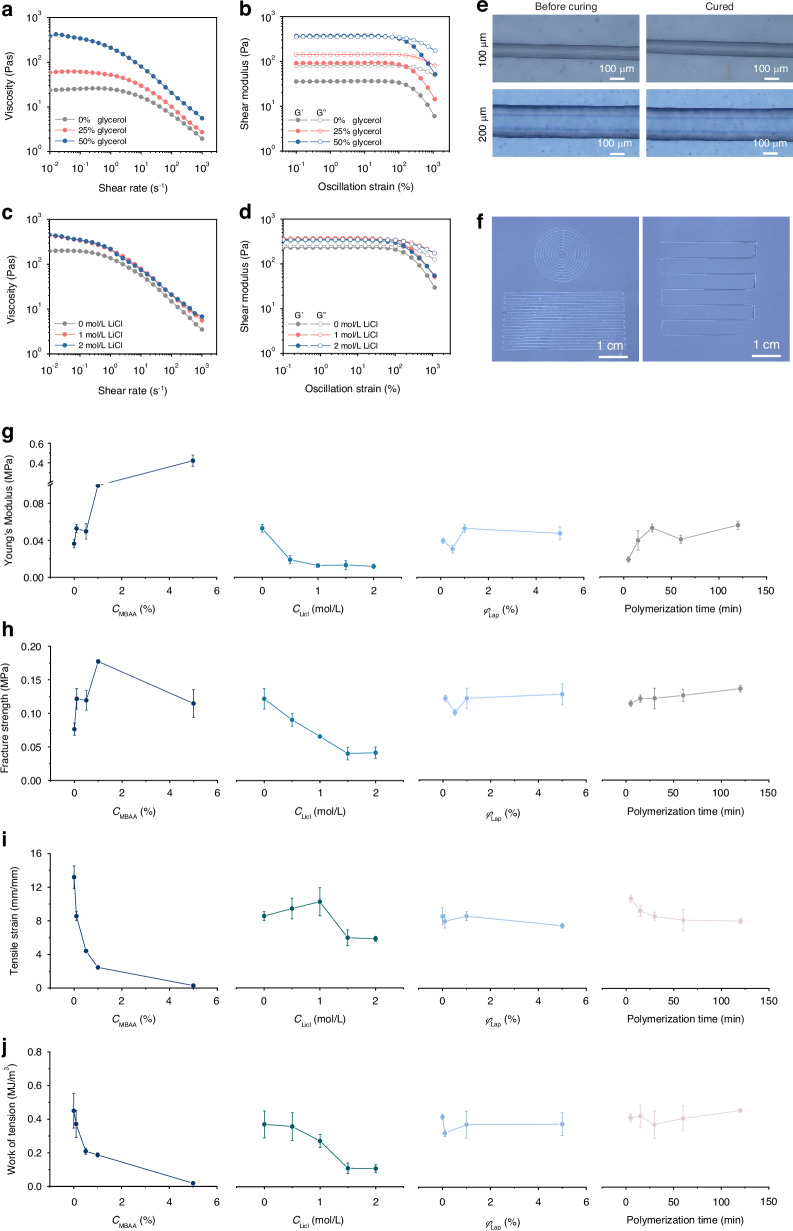
Rheological and mechanical properties of PIC-G ink. **a**, **b** Viscosity (**a**) and storage (*G*’) and loss (*G*”) moduli (**b**) of PIC-G inks by varying glycerin concentration from 0% to 50% by weight. **c**, **d** Viscosity (**c**) and storage (*G*’) and loss (*G*”) moduli (**d**) of PIC-G inks by varying LiCl concentration from 0 mol/L to 2 mol/L. **e**, **f** Photographs of 3D-printing PIC-G inks into different patterns using nozzles with different inner diameter; **g**–**j** Young’s modulus (**g**), Fracture strength (**h**), Tensile strain (**i**), and Work of tension (**j**) of PIC-G hydrogels as a function of gel compositions

The printability of the PIC-G ink was significantly influenced by the concentration of LiCl or glycerol. The rheological properties of the PIC-G ink were evaluated using different concentrations of LiCl (0, 1, and 2 mol/L) or glycerol (0, 25, and 50 wt%). Irrespective of the LiCl or glycerol content, all PIC-G inks exhibited shear-thinning behaviors (Fig. 2a, c), with shear modulus, storage modulus (*G*’), and loss modulus (*G*”) increasing as the concentration of LiCl or glycerol increased (Fig. 2b, d). As the amount of glycerol rises, the hydrogen bonding density within the gel network increases, resulting in a denser network structure. This tighter structure impedes the movement of solution molecule chains, thus elevating the viscosity of PIC-G inks (Fig. 2a). On the other hand, the presence of LiCl ions results in the formation of hydrated ions, surrounded by water molecules. This formation prompts water molecules to aggregate into larger structures, fostering enhanced interactions among them and ultimately leading to higher solution viscosity (Fig. 2c).

The viscoelastic properties of PIC-G inks were also affected by the concentration of LiCl or glycerol. The inks’ dynamic modulus, that is, storage modulus (*G*’), and viscous behavior, that is, loss modulus (*G*”), were assessed at different shear strain regions (Fig. 2b, d). With 0% or 25% glycerin, the ink exhibited a higher loss modulus than storage modulus (*G*” > *G*’) across all shear strain regions. This indicated a predominantly liquid-like behavior of the PIC-G ink, rendering it unavailable for high-resolution printing (Fig. 2b). Whereas, with a glycerol content of 50%, the ink displayed a more complicated behavior at different shear strain regions. At lower oscillation strains (below 100%), the higher storage modulus (*G*’ > *G*”) indicated the elastic behavior of the ink necessary for maintaining the structural integrity of the printed material. Conversely, at higher oscillation strain, such as 100%, the storage (*G*’) and loss (*G*”) moduli began to intersect, marking the onset of the ink’s elastic deformation. Eventually, at much higher oscillation strain of up to 1000%, the loss modulus surpassed the storage modulus (*G*” > *G*’), where the ink transferred into a liquid state, allowing its extrusion from the nozzle under air pressurization and subsequent recovery of elastic stability after printing completion (Movie S1). Besides, the viscoelastic properties of PIC-G inks were also presented in terms of dumping factor of Tan*δ* (*δ* = *G*”/*G*’) as a function of oscillation strain in Fig. S2. The rheological studies elucidated that an increase in shear rate led to the breakdown of local structures of hydrated ions, releasing entrapped liquid and resulting in reduced viscosity and shear-thinning behavior. This effect could be compensated by adding glycerol or LiCl to increase the ink’s viscosity sufficiently for inkjet printing, thereby maintaining the printed structures in the designed pattern.

The resolution of 3D printing is crucial for array fabrication and its spatial sensing capability. In general, ink resolution is influenced by the printing speed (V_p_), printing height (H), nozzle diameter (D), and air pressure (P). In our study, the effects of two main factors, P and V_p_, on the linearity of the printed structures were investigated using a LiCl-glycerol combination of 1 mol/L - 50 wt% of PIC-G ink. Specifically, we varied the printing speed at 1 to 8 mm/s, and conducted experiments under three different air pressures: 100, 200, and 300 kPa. Each condition was tested in triplicate. The diameter of each filament was observed and measured using an electron microscope. As shown in Figs. S3 and S4, the results indicate that, at a constant air pressure, the diameter of the printed filament decreases as the printing speed increases. Conversely, at a constant printing speed, the filament diameter increases with higher air pressure. These results are consistent with observations reported in other studies on 3D printing^47^. In addition, we noted that the interplay between air pressure and printing speed can be fine-tuned to achieve the desired filament width and resolution, highlighting the importance of optimizing these parameters for high-quality hydrogel printing. As shown in Fig. 2e, f, by fine-tuning the printing parameters such as pressure or speed, the widths of the printed gel strips could be precisely regulated to match the inner diameter of the nozzle.

Variations in mechanical properties between the electrode and the skin may impact the stability and precision of sEMG signals. Therefore, it is essential to tailor the mechanical characteristics of the PIC-G gel accordingly. Several parameters, such as cross-linker (MBAA) density, LiCl concentration, photoinitiator volume, and polymerization time, were analyzed to assess their effects on the mechanical attributes of the PIC-G gel. As illustrated in Fig. 2g–j, the PIC-G gel without MBAA exhibited a low Young’s modulus (*E* = 0.04 MPa), weak fracture strength (*σ*_b_ = 0.08 MPa), high tensile strain (*ɛ*_b_ = 13.19 mm/mm), and work of tension (*W*_b_ = 0.45 MJ m^−3^). When increasing MBAA to 5%, the gel became stiffer but more fragile, resulting in the former two mechanical properties rising to *E* = 0.42 MPa and *σ*_b_ = 0.12 MPa, while the latter two decreased to *ɛ*_b_ = 0.29 mm/mm and *W*_b_ = 0.01 MJ m^−3^. This can be attributed to the enhancement of cross-linking density within the hydrogel network due to increased MBAA content, thereby reinforcing intermolecular connections. On the contrary, the introduction of LiCl disrupts the original ionic bonding within the hydrogel, thus weakening intermolecular connections. Consequently, the relationship between PIC-G gels’ mechanical strengths and LiCl concentrations exhibits an exact inverse pattern compared to that with MBAA. For instance, both *E* and *σ*_b_ decreased from 0.05 MPa to 0.01 MPa and from 0.12 MPa to 0.041 MPa, respectively, with an increase in LiCl concentration from 0 mol/L to 2 mol/L. Conversely, both *ɛ*_b_ and *W*_b_ of the gel decreased with an increase in LiCl concentration, mirroring the trend observed with MBAA. On the other hand, neither photoinitiator concentration nor polymerization time significantly affected the gel’s mechanical properties. For instance, with an increase in photoinitiator volume from 0.1% to 5%, both *E* and *σ*_b_ maintained approximate values at 0.04 MPa and 0.013 MPa, respectively. Similarly, with increasing polymerization time from 5 to 120 min, both *E* and *σ*_b_ slightly increased from 0.02 MPa to 0.056 MPa and from 0.11 MPa to 0.136 MPa, respectively. This slight increase was attributed to the latter sharing a similar pattern with its effect on the gel’s mechanical properties as cross-link density does, since the longer the curing time, the denser the hydrogel structure. In summary, modifying the concentration of MBAA or LiCl has a greater impact on the mechanical adjustment of the gel compared to variations in photoinitiator volume or polymerization time.

**Fig. 3:**
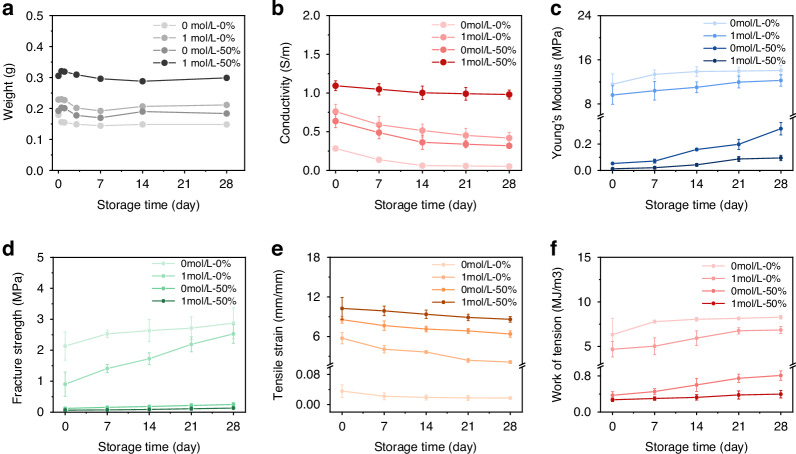
Long-term stability of PIC-G gels. **a** The weight of PIC-G hydrogel varying with the variation of gel formulations, revealing the smallest weight change at the LiCl-glycerol combination of 1 mol/L - 50 wt%. **b** Conductivity of PIC-G hydrogels as a function of time, revealing a stable conductivity over 28 days at the LiCl-glycerol combination of 1 mol/L - 50 wt%. **c**–**f** Young’s modulus (**c**), Fracture strength (**d**), Tensile strain (**e**), and Work of tension (**f**) in response to four types formulations of PIC-G hydrogels over 28 days drying

The prolonged durability of the PIC-G Gel as a human-machine interface ensures its stability and reliability over time. To assess their ability to uphold moisture, mechanical, and electrical properties, gels were subjected to routine evaluation over a 28-day period in a programmable constant temperature and humidity chamber at a constant temperature of 25 °C and relative humidity (RH) of 70%. Four different gel configurations were examined, varying in LiCl-glycerol concentrations: 0 mol/L - 0%, 0 mol/L - 50%, 1 mol/L - 0%, and 1 mol/L - 50%. As shown in Fig. S5, the presence of glycerol significantly influenced the gel’s water retention capacity compared to LiCl. Gels containing 50% glycerol with either 0 or 1 mol/L LiCl maintained their optical transparency throughout the testing period. Conversely, those with 0% glycerol and either 0 or 1 mol/L LiCl appeared to undergo a drying process, exhibiting a nearly opaque white color and reduced transparency. This was primarily attributed to glycerol’s pronounced hygroscopic nature, enabling it to form hydrogen bonds with water molecules. While Li^+^ ions have a weaker hygroscopic ability than glycerol, they still attract and bind water molecules for retention. Consequently, the combination of glycerol and LiCl at concentrations of 1 mol/L - 50% demonstrated the most effective water retention capabilities, maintaining a nearly constant weight of around 0.3 g over 1-month storage period, whereas slight weight loss was observed in all other configurations (Fig. 3a). Furthermore, compared to the former three configurations, the latter one with glycerol and LiCl at concentrations of 1 mol/L - 50% also exhibited prolonged consistent performances in its electrical and mechanical properties. For instance, at day 0, it presented an electrical conductivity of 1.09 S/m (Fig. 3b) and a set of mechanical strengths, including *E* = 0.013 MPa, *σ*_b_ = 0.064 MPa, *ɛ*_b_ = 10.26 mm/mm, and *W*_b_ = 0.272 MJ m^−3^ (Fig. 3c–f). These parameters remained relatively unchanged at day 28. Conversely, all other configurations experienced certain variations in either their electrical or mechanical characteristics. Overall, all samples demonstrated structural integrity, indicating their suitability for long-term usage.

### Myoelectric properties of PIC-G-Ag electrode

**Fig. 4:**
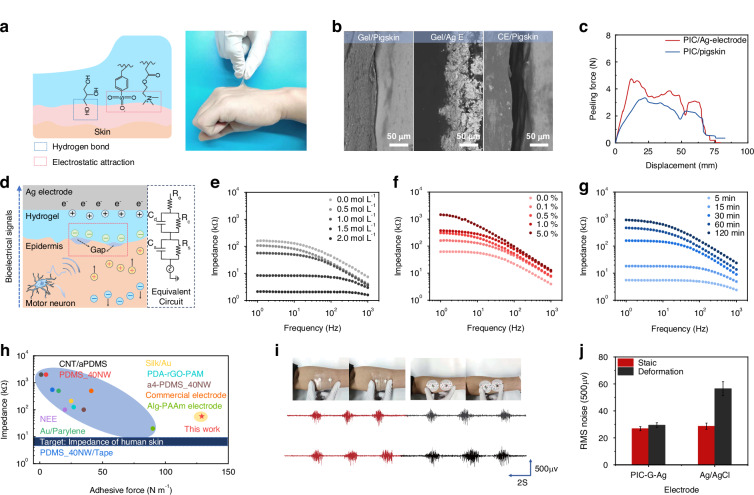
Myoelectric Properties of PIC-G-Ag electrode. **a** Adhesion mechanism and optical photograph of PIC-G gels attaching tightly on the skin. **b** SEM images of micrometer-scale gaps existing at the interface between commercial electrode and pigskin while no such gaps at the interface of PIC-G gel to pigskin as well as to Ag electrode. **c** Adhesion strengths of PIC-G gels against pigskin or Ag electrode. **d** Schematic of the electron-ion transducing from epidermis to electrode and the equivalent circuit model. **e**–**g** Interfacial resistance of PIC-G gels as a function of LiCl, MBAA, and photopolymerization time. **h** Comparison between this work and reported sEMG electrodes in terms of contact impendency and adhesive force, with the blue bar representing the impedance of human skin (6–10 kΩ). **i**, **j** sEMG signal quality of the PIC-G-Ag electrode under cyclic deformation in comparison to the commercial electrode

Motion artifacts pose a significant challenge to obtaining high-quality signals with sEMG electrodes. One effective solution involves achieving a tight adhesion and minimizing the spatial gap between the skin and the epidermal electrodes. To fulfill this requirement, this work employed an in situ polymerization of PIC-G gels on Ag electrodes via 3D printing. By utilizing PIC-G gels as binders, a robust interfacial connection between the Ag electrodes and the skin was established. The effectiveness of this approach was demonstrated in Fig. 4a, where the strong adhesion between the PIC-G gels and the skin was evident, as pulling the gel on the forearm resulted in the upward movement of a layer of epidermis rather than delamination from the skin. This robust adhesion is attributed to the synergistic effect of hydrogen bonding and electrostatic attraction between the gel and the skin. PIC-G hydrogels contain a significant amount of oppositely charged polyelectrolytes, allowing them to form electrostatic attractions with the skin when in close proximity. Based on the available research^46^, Polyampholyte Hydrogels (PA hydrogels) adhere to physiological surfaces through dynamic ionic bonds that form ionic complexes with induced polarization, creating attractive interaction sites. The strong adhesion of PA gels primarily stems from electrostatic interactions in an ionic equilibrium state. However, the PIC-G hydrogels exhibit even stronger adhesion due to the non-equilibrium state of the ions within the gel, resulting in more free ions being exposed and thus stronger adhesion compared to PA gels. In addition, we introduced glycerol into the PIC-G hydrogels, which increases the number of hydrogen bonds within the gel, significantly enhancing adhesion. The combination of electrostatic interactions and the increased hydrogen bonding from glycerol and LiCl contributes to the superior skin adhesion observed in our PIC-G hydrogels. Besides, scanning electron microscope (SEM) photographs revealed micrometer-scale gaps (~45 μm) between commercial electrode and pigskin (which has a structure similar to human skin) (Fig. 4b), while almost no gaps were observed at the interface of PIC-G gel with pigskin or Ag electrode, indicating its excellent tissue adhesion capability and skin-like modulus. Quantitative analysis of the gel’s interfacial mechanical strength was conducted through a 180° peeling test on the gel-to-pigskin and the gel-to-Ag electrode, yielding adhesion strengths of 129 and 177.4 N m^−1^, respectively (Fig. 4c). This indicates that PIC-G gel offers significant advantages in constructing a seamless and robust human-machine interface.

Figure 4d shows the coupling process between ion fluxes in PIC-G hydrogel and electron currents in Ag electrode, along with their equivalent circuit model. When a muscle is activated, a sEMG signal is generated and travels through the epidermis to the hydrogel and subsequently to the Ag electrode in series, completing the acquisition of the sEMG signal. However, muscle contraction usually results in the formation of micrometer-sized or larger gaps between the electrode and the curved skin. The electrode-skin mechanical mismatch disrupts the capacitive transduction of the electron-to-ion process, leading to a decrease in the induced charge density at the electrode-skin interface and an increase in interface impedance. This could ultimately introduce electromechanical noise and distort the quality of sEMG signals.

Several factors, such as LiCl, MBAA, and polymerization time, were examined at their effects on the myoelectric properties of PIC-G gels as electrode-skin interfaces. As previously indicated (Fig. 2g), the Young’s modulus of PIC-G gels decreased with increasing LiCl content, which can reduce the physical gap between the electrode and the skin, thereby increasing electrode compliance with the skin and decreasing interfacial impedance. Simultaneously, the dialysis of LiCl into Li^+^ and Cl^-^ ions in gel network elevated the ionic conductivity of the gel (Fig. S6). Figure 4e demonstrated the combined effects of increasing LiCl concentration from 0 to 2 mol/L resulting in a two-order decrease in interfacial impedance. In contrast, the effect of cross-linker (MBAA) on gel electrical properties exhibited an inverse pattern compared to that of LiCl. Increasing the MBAA density from 0 to 5% led to a two-order increase in interfacial impedance (Fig. 4f). This is because the increased MBAA tightens and stabilizes the hydrogel network, limiting the free movement of ions and reducing electrical conductivity, which causes the increase of interfacial impedance. Similarly, the relationship between polymerization time and interfacial impedance showed that a longer polymerization time resulted in higher interfacial impedance, as increased polymerization time leads to a corresponding increase in cross-linking reactions, resulting in a denser hydrogel network but higher interfacial resistance (Fig. 4g). Overall, the PIC-G gel possessed strong adhesion with an adhesive force of 128.97 N m^−1^ and a contact impedance of 56.79 kΩ, which closely approximates the ideal impedance range of 6–10 kΩ for an electrode-skin system at a working frequency of 1 Hz^48^. These characteristics surpass those reported in most literature (Fig. 4h, Table S1).

The durability of the assembled electrode, referred to as the PIC-G-Ag electrode, against motion artifacts was assessed by examining its sEMG quality during a grasping exercise with 50% maximal voluntary contraction (MVC) and simultaneous cyclic stretching and compression of the skin around the electrodes. A commercial Ag/AgCl electrode underwent the same procedure for comparison purposes. As depicted in Fig. 4i, manual deformation of the surrounding skin caused the Ag/AgCl electrode to shift from its original position, while the PIC-G-Ag electrode maintained a snug fit with the skin. A quantitative analysis comparing the baseline noise RMS (root mean square) and the signal-to-noise ratio (SNR) values of Ag/AgCl electrodes and PIC-G-Ag electrodes was conducted. When the skin was at rest, the RMS value recorded at the PIC-G-Ag electrode was 26.89 µV, slightly lower than the 29.47 µV measured at the Ag/AgCl gel electrode. Conversely, during skin stretching or compression, the PIC-G-Ag electrode consistently maintained an RMS noise value of 28.65 µV, while that of the Ag/AgCl electrode doubled to 56.47 µV (Fig. 4j). Correspondingly, as shown in Fig. S7, the changes in SNR values during this process reflect variations in signal quality. In a stationary state, the SNR of the PIC-G-Ag electrode was 3.34 dB higher than that of the Ag/AgCl electrode. During movement, the SNR of the PIC-G-Ag electrode remained relatively stable, while the SNR of the Ag/AgCl electrode significantly decreased. This indicates that the strong adhesion and myoelectric stability of the PIC-G-Ag electrode allow it to withstand interference from motion artifacts, maintaining a low level of baseline noise even in the presence of significant skin deformation and ensuring better signal quality.

### Biocompatibility of PIC-G electrodes

To assess the biocompatibility of the PIC-G electrode, we co-cultured the PIC-G hydrogel and PIC-G-Ag electrode with human immortalized keratinocytes (HaCaTs) for 24 and 72 h, respectively, to evaluate cell viability. As shown in Fig. S8, over 99% of the cells remained viable on both the PIC-G hydrogel and PIC-G-Ag electrode after 24 h. After 72 h of incubation, cell viability remained stable, with no statistically significant difference observed in the survival rate between the PIC-G hydrogel, PIC-G-Ag electrode, and the control group. The results demonstrate that the PIC-G-Ag electrode exhibits good biocompatibility with human skin cells, particularly showing no signs of acute toxicity. In addition, the design of the electrode, in which the silver layer is sandwiched between the hydrogel and a polyurethane substrate, effectively prevents the silver from leaking into surrounding tissues. This structural feature minimizes potential cytotoxicity, further enhancing the biocompatibility of the assembled electrode and confirming its suitability for a broad range of dermal applications.

### Smart control of prosthetic hand

**Fig. 5:**
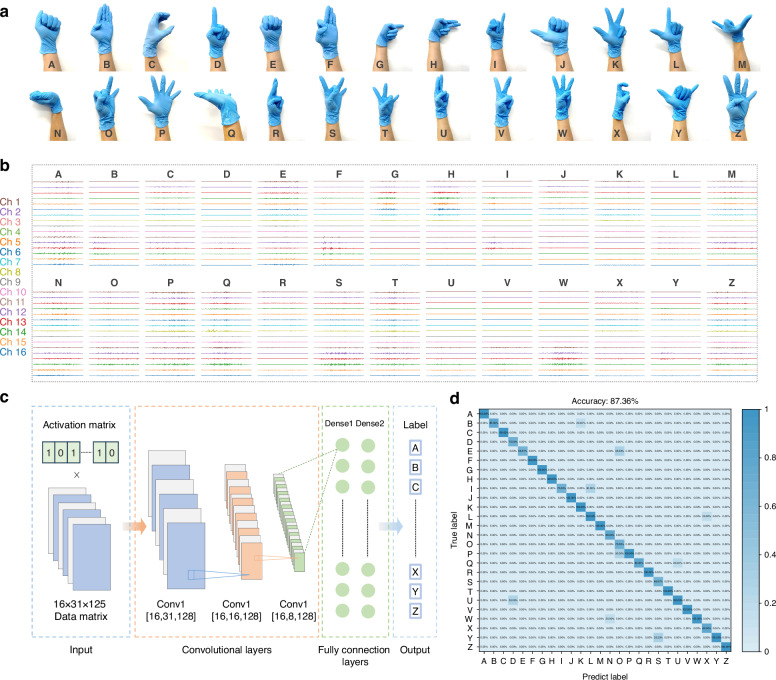
Smart hand gesture identification. **a** Photographs of 26 gestures, **b** with their raw sEMG data via a 16-channel readout. **c** Schematic illustration of the trained CNN algorithm. **d** Confusion matrix of the recognition of 26 gestures

Smart control of a prosthetic hand was executed through hand gesture recognition using a wearable, wireless sEMG acquisition system comprising the PIC-G-Ag electrode and a 16-channel analog readout. The experiment involved performing flexion and extension movements of various fingers across different degrees of freedom, corresponding to the gestures representing the 26 letters of the English alphabet (Fig. 5a). Four healthy participants were enlisted, and the device was positioned with the electrode center roughly aligned with the ulna of their dominant arm (see sEMG Signal acquisition for hand gestures in the “Experimental section” for details).

Figure 5b depicted the raw waveforms captured from all 16 channels during the flexion and extension of 26 gestures. Notably, the signals collected from each volunteer performing the A action show individual differences. This variation is anticipated, given the differences in muscle structure and skin surface conditions among individuals (Fig. S9). Prior to being fed into a machine learning network for training, the raw data underwent preprocessing, mainly filtering. The preprocessing steps included applying a bandpass filter ranging from 20 to 500 Hz, a series of 50 Hz power frequency notch filters with multipliers, and performing wavelet noise reduction sequentially. Following this, a 125 ms window was applied to segment the data with a 62.5 ms overlap between consecutive segments. As a result, 31 segments were created, each containing 125 sampling points. Segments exhibiting an averaged amplitude greater than 30% of the maximum among the 31 segments were considered active. Ultimately, these procedures generated input data with dimensions of 16 × 31 × 125 for the network, which utilized a convolutional neural network (CNN) framework—an algorithm developed in our previous work to recognize swallowing activities from 16-channel sEMG data^49^ (Fig. 5c). The CNN architecture comprised three convolutional layers, accompanied by two pooling layers and two fully connected layers. The convolutional layer contains residual blocks that utilize previous feature information to further optimize and enhance the features currently being extracted, thus improving the performance of the model; while the pooling layer reduces the dimensionality of the data to eliminate redundancy and mitigate the risk of overfitting. Subsequently, the fully connected layers, each comprising 512 nodes, employ regression methods to classify the features and subject them to nonlinear transformations to map them into vectors. For the classification of 26 hand gestures, a softmax layer was utilized to map the 512-dimensional vectors to a vector of length 26. It’s worth noting that during model training, the sEMG data from various subjects were randomized, a strategy intended to enhance the model’s ability to generalize unseen data. Figure S10 displayed the t-distributed Stochastic Neighbor Embedding (t-SNE) scatterplots of the 26 hand gestures. Following dimension reduction, 26 unique clusters with distinct boundaries were generated. This indicates that machine learning algorithms can effectively discern differences in signal data across various gestures. Therefore, it is reasonable to infer that CNN can attain high classification accuracy. The results exhibited a high average classification accuracy of 87.36% by using this model (Fig. 5d).

**Fig. 6:**
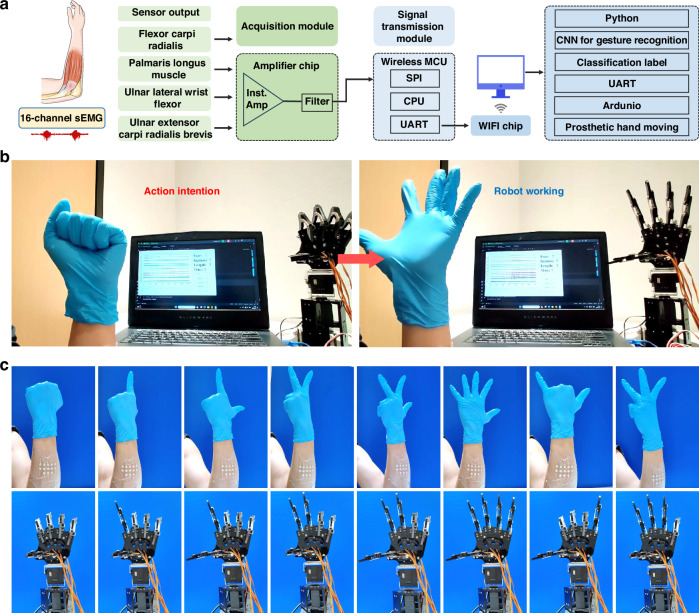
The demonstration of real-time, high-precision control of the prosthetic hand. **a** Working flow chart and **b**, **c** photographs showing a prosthetic hand executing a set of hand gestures via the system

The fusion of sEMG electrodes with a trained CNN algorithm facilitates intelligent control of a robotic hand, offering potential benefits in assisting physically disabled individuals with basic daily tasks and promoting social engagement through prosthetics. To enable this functionality, the sEMG patch must be securely affixed to the target muscles of the subject’s forearm, encompassing muscles such as Flexor carpi radialis, Palmaris longus muscle, UInar lateral wrist flexor, UInar extensor carpi radialis brevis. The gestures performed, as indicated by collected sEMG signals, undergo a series of hardware and software processing steps, obtaining their labels within the CNN framework. These labeled signals are then utilized to guide a prosthetic hand in replicating the corresponding gestures (Fig. 6a). To illustrate this capability, the control process based on this system was evaluated through its application on a volunteer. As depicted in Movie S2 and Fig. 6b, when the volunteer simulated a physically disabled individual by making a weak fist gesture, the prosthetic hand accurately replicated the gesture upon receiving the decoded signal. Subsequently, when the volunteer expressed an intent to release the fist, the prosthetic hand promptly opened all five fingers. Notably, the method of interactive control exhibited commendable accuracy and repeatability, with a response time of ~3 s following the volunteer’s active movement intention. Real-time, high-precision control of a prosthetic hand was successfully achieved as volunteers performed various gesture movements (Fig. 6c), showcasing promising application prospects and practical significance.

## Conclusions

Here, we have developed a polyion complex glycerol hydrogel, termed as PIC-G hydrogel, which is suitable for 3D printing and allows for adjustment of its mechanical and electrical properties by altering its composition. By employing layer-by-layer printing and lamination techniques, we crafted a stretchable, flexible, and high-density sEMG electrode array. This electrode array possesses several desirable HMI characteristics, including sustained functionality for nearly 1-month, a low bioelectrical interfacial impedance of 56.79 kΩ, and a robust adhesion force of 128.97 N m^−1^. When integrated with an external readout circuit, the system enables its compactness, light weight, and wearing comfort, and does not pose any additional burden or risk of injury to the user. Utilizing a well-designed CNN network, sEMG data can be accurately and efficiently decoded and transmitted to a prosthetic hand via the system. In summary, this system is capable of precisely discerning motion intentions and enabling physically disabled individuals to perform basic daily activities using a prosthetic hand.

## Experimental section

### Materials

Lithium chloride (LiCl), sodium p-styrenesulfonate (NaSS, 90 wt%), and glycerol (C_3_H_8_O_3_, 99 wt%) were procured from Sigma-Aldrich. Lithium Phenyl (2,4,6-trimethylbenzoyl) Lithium phenyl-2,4,6-trimethylbenzoylphosphinate (Lap, 98%) and Dimethylaminoethylacrylate quaternized ammonium (DMAEA-Q, 80 wt%) were obtained from J&K Chemical Ltd. α-ketoglutaric acid (α-keto) and *N,N’*-methylene-bis-acrylamide (MBAA) were sourced from Sinopharm Chemical Reagent Co. Ltd. All these reagents, which are of analytical grade, were used as received. For all experiments, deionized water (DI, 18.3 MΩ) was employed.

### Preparation of PIC-G gel

#### Synthesis of PNaSS powder

Specifically, in the case of 70 °C water bath and deionized water as a solvent, a homogeneous prepolymerization solution was prepared containing 1 M sodium *p*-styrenesulfonate (NaSS, 90 wt%) in the presence of 0.05 mol% *α*-ketoglutaric acid (*α*-keto). Then, the pre-polymerized solution was quickly injected into a reaction cell consisting of a pair of glass plates as walls and a silica gel gasket (10 cm × 10 cm × 0.1 cm). The reaction cell was then irradiated by an ultraviolet lamp (405 nm, 10 W cm^−2)^ for polymerization for 10 h at ambient temperature, to form pre-hydrogels. The pre-gel was dried in an oven at 65 °C for 24 h and then ball-milled at 320 revolutions per minute, which was conducted by three times. Finally, uniformly PNaSS powder was obtained through a 300-mesh sieve.

#### Preparation of PIC-G ink

PIC-G hydrogel was prepared by a free radical polymerization. Typically, the anionic PNaSS powder, cationic monomer (DMAEA-Q), cross-linker (MBAA), and photoinitiator (Lap) was prepared. The molar fraction of the anionic monomer was fixed at 0.5, and the total ionic monomer concentration (*C*_M_) was 2.3 mol/L. In addition, the molar fractions of both the cross-linker and the photoinitiator were maintained at 0.1 mol%, relative to *C*_M_. The specific steps are as follows: The prepared PNaSS powder and glycerol solution at 70 °C water temperature water bath are heated and stirred for 10 min to get a viscous solution, and then added with the cationic monomer DAMEA-Q, cross-linking agent MBAA, photoinitiator (Lap), as well as LiCl powder in a 70 °C water bath to get the PIC-G ink via 10 min heating and stirring.

### 3D printing of PIC-G gels

The 3D printing of the PIC-G gel was carried out by Power Square’s MP series microelectronic printer platform. During the printing process, the PIC-G ink after removing air bubbles is loaded into a syringe and connected through a nozzle. To print PIC-G gel in a desirable pattern with high resolution, various parameters, such as air pressure, nozzle size, distance between nozzle and substrate, and nozzle speed, require a careful adjustment. Following printing, the gel is irradiated by 30 min through an ultraviolet lamp (405 nm, 10 W cm^−2^) for a complete polymerization.

### Mechanical characterization

#### Tensile tests

Uniaxial tensile test was conducted using a universal testing machine (CMJ4000, China) equipped with a 5 kN load cell. Prior to the test, the hydrogel sample is meticulously cut into dumbbell-shaped specimens with a gauge length (l) of 12 mm, width (w) of 2 mm, and approximate thickness (t) of 1 mm. These tests are carried out at ambient temperature and a constant stretching velocity of 100 mm/min. The Young’s modulus of the hydrogels is determined by calculating the initial slope of the stress-strain curves within a strain limit of 10%. In addition, the work of tension is quantified by integrating the area under the stress-strain curves:

1 $${W}_{{\rm{t}}}={{\int}_{0}}^{{\varepsilon }_{{\rm{b}}}}\sigma d\varepsilon$$

where *σ* and *ε* are the stress and strain, respectively, and ${\varepsilon }_{\text{b}}$ is the strain at breaking of the samples.

#### Adhesion test

The interfacial toughness of PIC-G gel in contact with pigskin was evaluated using the 180-degree peel protocol as per the ASTM F2256 standard. The bonding strength is determined by calculating the average of platform forces during a stable peeling state and dividing it by the adhesion width. Each sample has a dimension of 50 mm in length, 15 mm in width, and 2 mm in thickness. To prevent stretching of the hydrogel samples during the adhesion test, they are bonded to poly (ethylene terephthalate) (PET) film using cyanoacrylate adhesive. Prior to testing, a 5 N load is applied to each sample for 60 s. All tests are carried out at room temperature using a universal testing machine at a consistent tensile speed of 100 mm/min.

### Rheological characterization

#### Rheological test

The rheological properties of PIC-G inks were analyzed using a rotational rheometer (DHR; TA Instrument), equipped with a steel of 40-mm diameter parallel-plate geometry. Viscosity is determined by analyzing shear rate during steady-state flow tests, covering a logarithmic range from 0.01 to 1000 s^−1^. All rheological assessments are conducted at a constant temperature of 25 °C, following a preliminary equilibration period of 1 min to ensure stability of the measurement.

### Electrical characterizations of PIC-G-Ag electrode

Electrode-skin impedance was assessed using an electrochemical analyzer (CHI627D, Shanghai Chenhua, China) equipped with a three-electrodes system. During the measurement, the reference and counter electrodes are positioned 3 cm and 6 cm away, respectively, from the working electrode, that is, the PIC-G-Ag electrode. Frequency scanning is conducted over a range from 1 to 1000 Hz to encompass the primary spectrum of sEMG signals. For long-term stability tests, the hydrogel electrodes are maintained in a programmable constant temperature and humidity chamber set at 25 °C and 70% humidity when not undergoing experimental testing. Single electrode sEMG data acquisition is conducted using the commercial Noraxon wireless sEMG acquisition system. The SNR (signal-to-noise ratio) is calculated as follows:

2 $${SNR}({db})=20\times {\log }_{10}\frac{\sqrt{{\sum }_{k=1}^{N}{V}_{{singal}(k)}^{2}}}{\sqrt{{\sum }_{k=1}^{N}{V}_{{noise}(k)}^{2}}}$$

### Biocompatibility characterization

Initially, human immortalized keratinocytes (HaCaTs) in the logarithmic growth phase were digested and prepared into cell suspensions, with the cell density adjusted to 10^5^ cells/mL using complete medium. Then, 100 μL of the cell suspensions were seeded into each well of a 96-well plate and incubated at 37 °C in a 5% CO_2_ atmosphere for 12 h. Following incubation, the original medium was replaced with medium containing different concentrations of the sample extracts, and the cells were incubated for an additional 24 or 72 h. At the end of the incubation period, the medium was removed, the wells were rinsed twice with (Phosphate-Buffered Saline) PBS, and 100 μL of Cell Counting Kit-8 (CCK8) working solution was added to each well. The plates were then incubated for 2 h at 37 °C, protected from light. Absorbance was measured at 450 nm using a microplate reader. The control group consisted of wells containing both medium and cells, while the blank group contained only medium and CCK8. Cell viability was calculated using the following formula: Relative cell viability (%) = (A_sample_ − A_blank_)/(A_control_ − A_blank_) × 100%.

### The recognition of hand gestures

16-channel array sEMG signals were gathered from 4 physically fit participants aged 19 to 25 years, all seated in a neutral position. The experimenter, maintaining the same seated position, prepared the skin of the arm area (including the Flexor carpi radialis, Palmaris longus muscle, UInar lateral wrist flexor, UInar extensor carpi radialis brevis) by cleaning it with a moist alcohol cotton ball. After waiting for the alcohol to evaporate and ensuring that the skin was dry, the experimenter proceeded to wear the electrodes on the hand to be tested. The fit of the electrodes to the skin was then inspected to minimize motion artifacts. Subsequently, following the gesture movements depicted in Fig. 5a, 25 repetitive sampling movements were performed for each gesture consecutively, with each action completed within a 2-s timeframe. The quality of the completed gestures was also verified using the host computer, with a 10-s interval between each repetition of the gesture action and a 60-s rest between gestures.

## Supplementary information

supporting information

Video S1

Video S2
