# Supplementary material for: 3D printable and myoelectrically sensitive hydrogel for smart prosthetic hand control
Source: Microsyst Nanoeng. 2025 Jan 21;11:15. doi: 10.1038/s41378-024-00825-y (PMC11747008; doi:10.1038/s41378-024-00825-y)
Supplement: Supplementary file 1 — supporting information [file 41378_2024_825_MOESM1_ESM.docx]

**Supporting Information**

3D Printable and Myoelectrically Sensitive Hydrogel for Smart Prosthetic Hand Control

Jinxin Lai^a^, Longya Xiao^a^, Beichen Zhu^a^, Longhan Xie*^a^, Hongjie Jiang*^a^


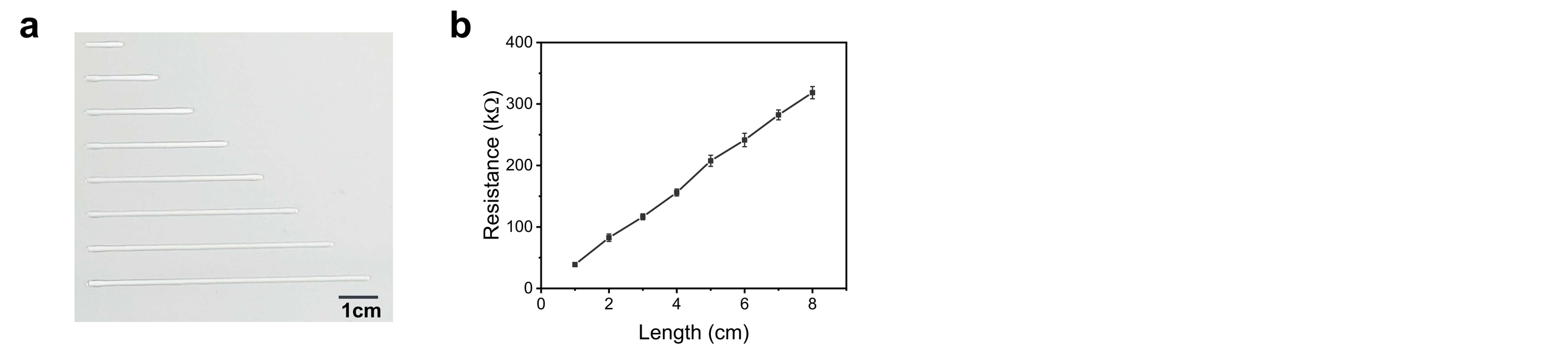


**Figure S1**. Uniformity demonstration of PIC-G inks. a) Photographs of eight strips from 1 centimeter to 8 centimeters via 3D printing with the ink and b) their resistances as a function of their lengths.


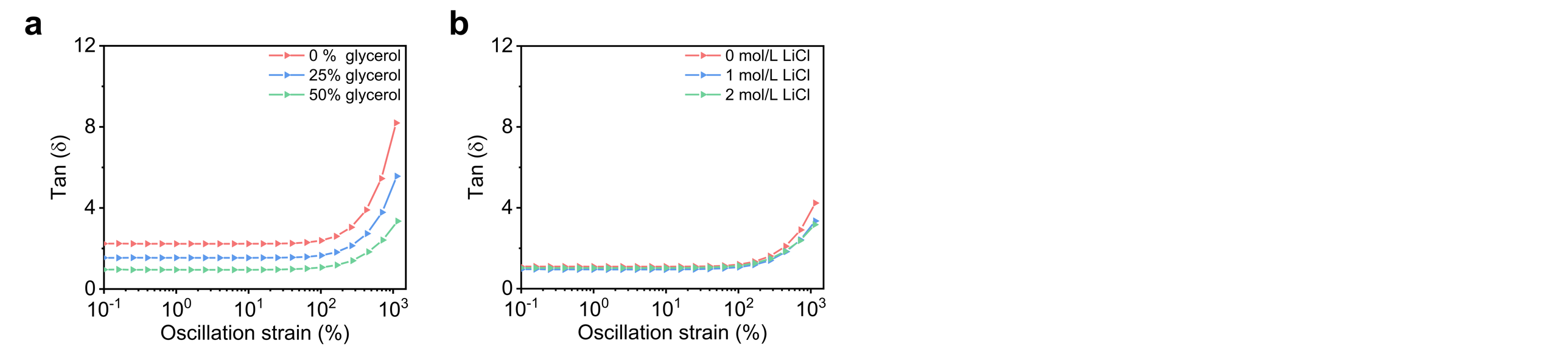


**Figure S2.** Tanδ (δ = *G*”/*G*’, with *G*” being the loss modulus and *G*’ being the storage modulus) as a function of the cyclic strain, varying at a) different glycerol volumes of 0, 25, or 50 wt% and at b) different LiCl concentrations of 0, 1, or 2 mol/L LiCl, varies. Tanδ > 1 indicating a liquid-like behavior of the ink while tanδ < 1 exhibiting a solid-like behavior. The results show that PIC-G inks with 50 wt% glycerol or 1 mol/L LiCl exhibit liquid-solid transition but liquid-like behavior with other configurations.


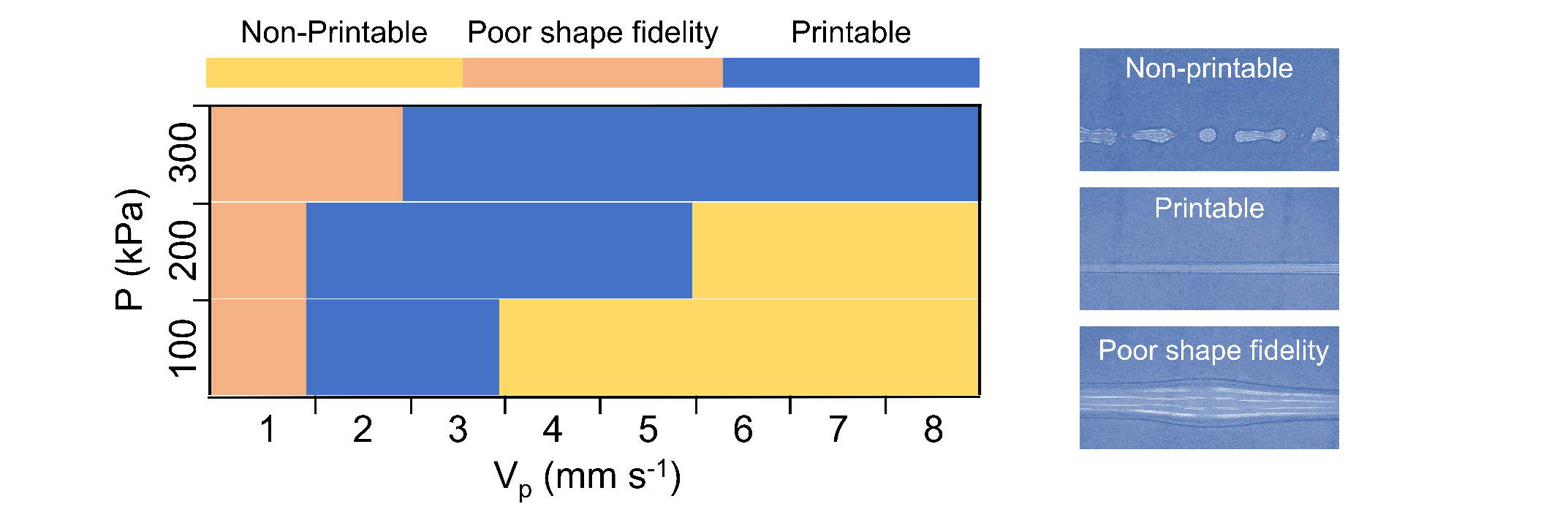


**Figure S3.** Phase diagram illustrates the printability of PIC-G ink under varying printing parameters. The printability region for the two materials is categorized into three color zones: orange, yellow, and blue, based on filament breakage and diameter distortion. When the applied pressure significantly exceeds the critical threshold or the printing speed is too slow, significant die swelling occurs, resulting in low-resolution prints. Conversely, low air pressure combined with high printing speed may lead to filament breakage.


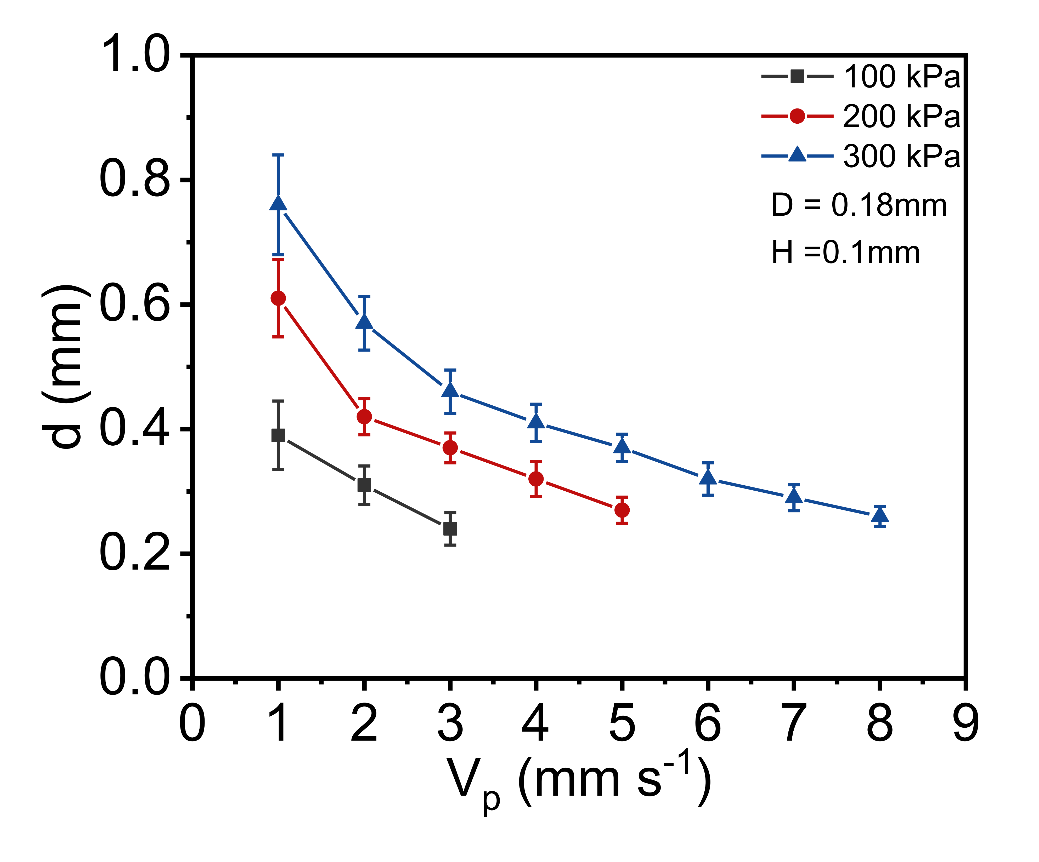


**Figure S4.** The relationship between the diameter of PIC-G filaments and printing speed was examined under various pressures (100, 200, and 300 kPa). The printing height was adjusted to 0.18 mm, using a 0.1 mm nozzle.

**
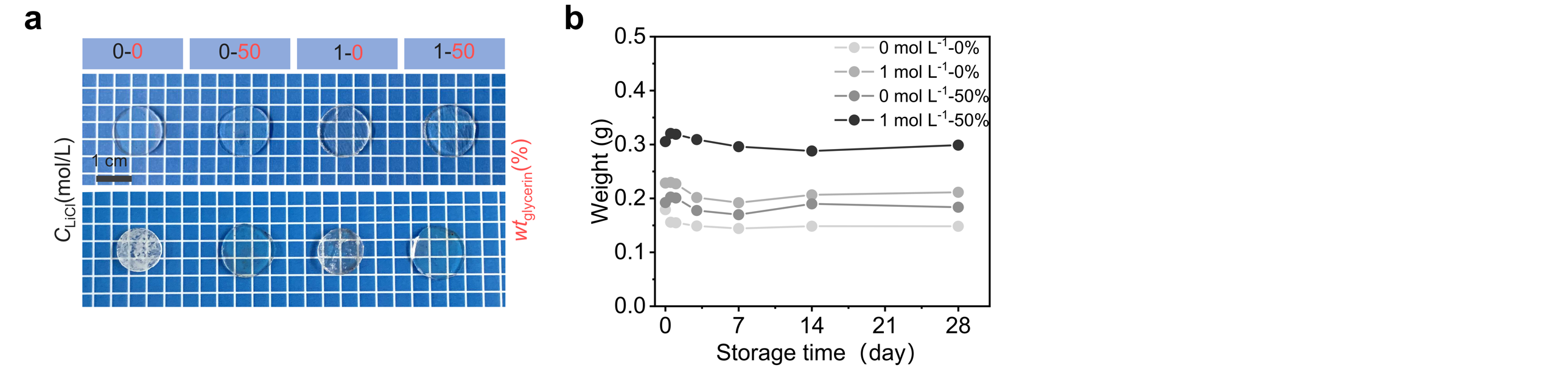
**

**Figure S5**. Long-term stability of PIC-G gels. Photographic demonstrations of PIC-G gels drying at room temperature over 28 days (top subfigures at day 0 and bottom subfigures at day 28)

**
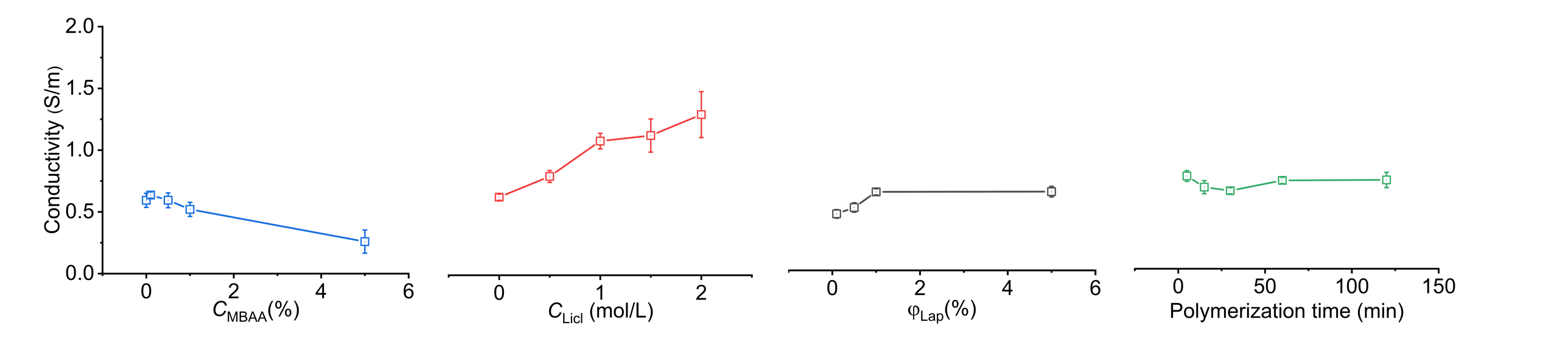
**

**Figure S6.** The variation of conductivities of PIC-G hydrogels by varying their formulations.


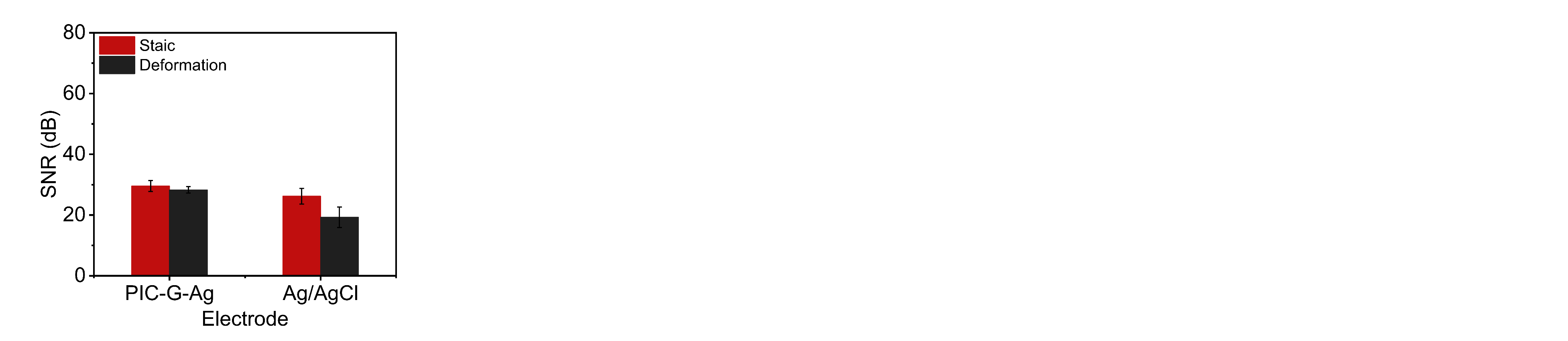


**Figure S7**. The SNR of the PIC-G-Ag electrode under cyclic deformation in comparison to the commercial electrode.


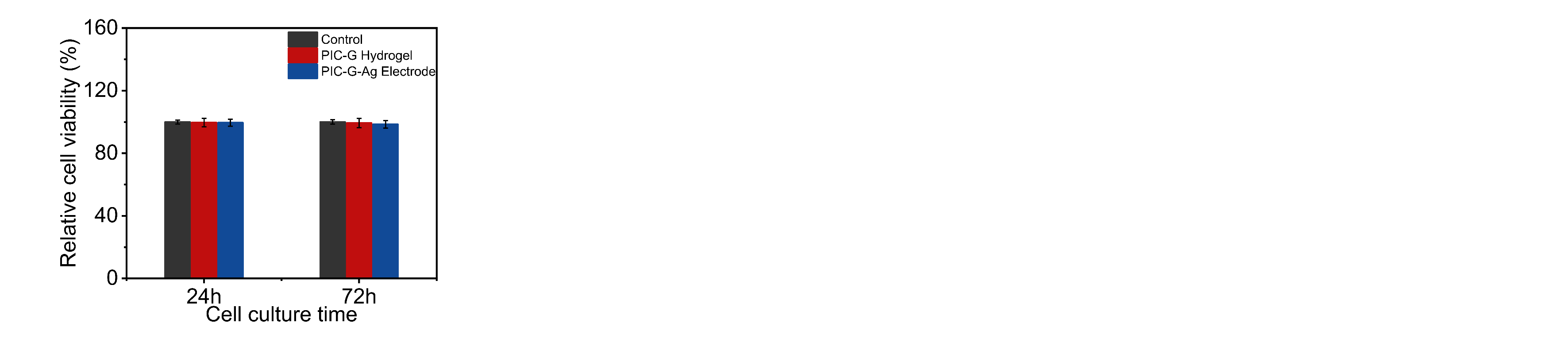


**Figure S8**. Relative cell viability (Rv) of cells cultured with PIC-G Hydrogel, PIC-G-Ag electrode, and control.


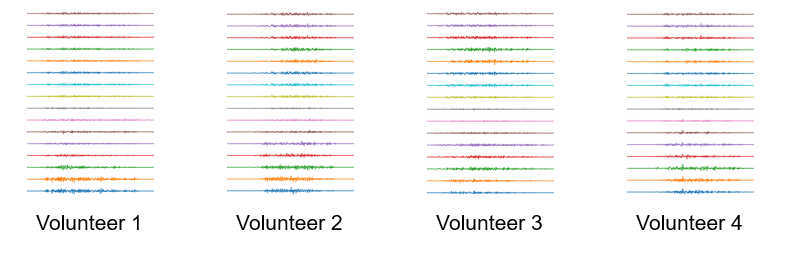


**Figure S9**. sEMG signals from four volunteers performing the A gesture.

**

**

**Figure S10.** t-SNE scatter plots for 26 gestures, where the sEMG data underwent dimensionality reduction to two dimensionless parameters.

**Supplementary Movies**

Supplementary Movie 1. Demonstration Video of PIC-G Ink for 3D Printing

Supplementary Movie 2. Demonstration video of a volunteer controlling a prosthetic hand using the sEMG system
